# An automated method for the analysis of food intake behaviour in *Caenorhabditis elegans*

**DOI:** 10.1038/s41598-018-21964-z

**Published:** 2018-02-26

**Authors:** Mª Jesús Rodríguez-Palero, Ana López-Díaz, Roxane Marsac, José-Eduardo Gomes, María Olmedo, Marta Artal-Sanz

**Affiliations:** 1 Andalusian Center for Developmental Biology, Consejo Superior de Investigaciones Científicas/Junta de Andalucía/Universidad Pablo de Olavide, Departament of Molecular Biology and Biochemical Engineering, Carretera de Utrera, km 1, 41013 Seville, Spain; 2 Institut de Biochimie et Génétique Cellulaires – C.N.R.S. UMR 5095 and Université de Bordeaux, 1, rue Camille Saint-Saëns, 33077 Bordeaux Cedex, France; 3 Department of Genetics, University of Seville, Avenida Reina Mercedes s/n, 41012 Seville, Spain

## Abstract

The study of mechanisms that govern feeding behaviour and its related disorders is a matter of global health interest. The roundworm *Caenorhabditis elegans* is becoming a model organism of choice to study these conserved pathways. *C. elegans* feeding depends on the contraction of the pharynx (pumping). Thanks to the worm transparency, pumping can be directly observed under a stereoscope. Therefore, *C. elegans* feeding has been historically investigated by counting pharyngeal pumping or by other indirect approaches. However, those methods are short-term, time-consuming and unsuitable for independent measurements of sizable numbers of individuals. Although some particular devices and long-term methods have been lately reported, they fail in the automated, scalable and/or continuous aspects. Here we present an automated bioluminescence-based method for the analysis and continuous monitoring of worm feeding in a multi-well format. We validate the method using genetic, environmental and pharmacological modulators of pharyngeal pumping. This flexible methodology allows studying food intake at specific time-points or during longer periods of time, in single worms or in populations at any developmental stage. Additionally, changes in feeding rates in response to differential metabolic status or external environmental cues can be monitored in real time, allowing accurate kinetic measurements.

## Introduction

The regulation of food intake is a critical mechanism with major physiological impacts. On the one hand, the rate of food intake determines the rate of growth, and anomalous feeding behaviours are associated with an assortment of chronic diseases such as obesity, type 2 diabetes, cardiovascular diseases and some cancers^1–3^. On the other hand, dietary restriction has implications and beneficial effects on health and longevity^4–6^. Hence the growing interest and the increasing efforts to understand the processes and mechanisms that control feeding actions and its anomalies.

Despite their evolutionary distance, metabolism and central signalling pathways are highly conserved between mammals and *Caenorhabditis elegans*, and the regulation of feeding is not an exception^7^. Human and *C. elegans* genomes show a relevant orthology degree^8^. At least 50% of *C. elegans* genes have a human ortholog, and approximately 70% of genes related with human diseases have homologs in *C. elegans*^9^. Therefore*, C. elegans* has emerged as a powerful model to study food-related behaviours, shedding light in the underlying factors and mechanisms involved in the genetic regulation of feeding^10–15^.

*C. elegans* feeding occurs as a consequence of two motions: the cyclic contraction of the pharynx (pumping) and the isthmus peristalsis, being the former the better comprehended of the two^16^. These feeding motions can be modulated by the internal state of the worm and by environmental cues. Feeding rate is proportional to the worm pharyngeal pumping where bacteria are sucked from the environment and grinded up. Thanks to the transparency of *C. elegans* the grinder movement can be directly observed under a stereoscope. This allows the routine study of food intake by means of direct quantification of pumps per minute^17^. Alternatively, it can be indirectly measured using bacteria expressing fluorescent proteins or BODIPY dye^18^. While these methods are easily implemented, they are short-term and unsuitable for independent measurements of high numbers of animals. Therefore, it is challenging to find automated methods that allow continuous and long-term measurements to study food intake behaviour in a sizable number of individuals. Some methods based on food clearance assays, time-lapse imaging or electrophysiological readouts have been reported^19–21^. All these approaches cover some of these aspects; but automatic and continuous methods, sensitive enough to study food intake behaviour in both individual animals and populations, are essential and still lacking.

Here we present a high-throughput method to continuously measure food intake behaviour in single worms and in worm populations. This new approach is based on a method, previously described by some of us, that uses bioluminescence for continuous monitoring of development in *C. elegans*^22^. We measured bioluminescence in strains that constitutively and ubiquitously express luciferase (LUC)^23,24^. LUC catalyzes the conversion of the substrate luciferin into oxyluciferin, in a reaction that emits light^25^. The luminescence signal increases throughout development, but is abruptly interrupted coinciding with the lethargy characteristic of the molt stages^22^. The decrease in the signal is due to the specific depletion of ingested luciferin during the quiescent periods, what led us to hypothesise that the bioluminescence signal could report for food intake behaviours. Here, we validate the method for analysis of food intake using mutants with defective pharyngeal pumping, changes in environmental conditions, as well as drugs that increase or decrease pumping rates. As proof of principle we were able to detect a food intake defect in a mutant previously unsuspected of presenting this phenotype. This method is automated, can be performed in multi-well plates, and allows continuous monitoring of food intake and accurate kinetic measurements. The method is suitable for measuring both individual animals and small populations, making thus feasible screening a relatively large number of animals.

## Results and Discussion

### Luminescence assay for the analysis of food intake behaviour in *C. elegans*

**Figure 1:**
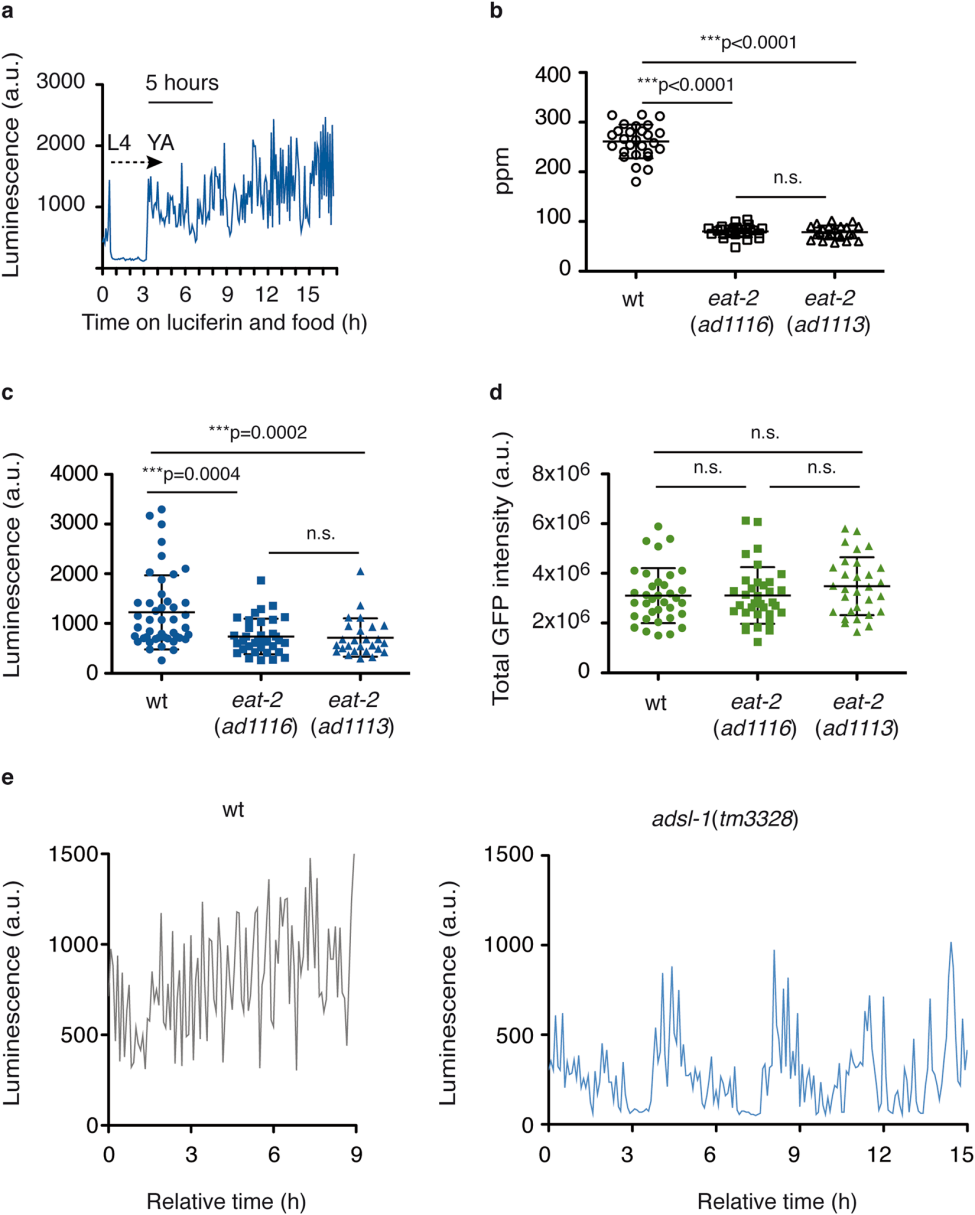
Luminescence signal reports food intake of single animals. (**a**) Bioluminescence signal from a single LUC::GFP transgenic animal over 17 h from the end of the L4 larval stage (t = 0). The absence of substrate intake during the molt provokes a decrease in the bioluminescence signal that resumes when animals exit the fourth molt and enter the young-adult stage (YA). The first 5-hour period of adulthood, used as a reference to calculate the mean value of the luminescence signal, is shown. (**b**) Pharyngeal pumping per minute (ppm) of wild-type, *eat-2*(*ad1116*) and *eat-2*(*ad1113*) mutant animals transgenic for LUC::GFP, at the YA stage. The graph shows data from two experimental replicates. (**c**) Mean luminescence signal of wild-type, *eat-2*(*ad1116*) and *eat-2*(*ad1113*) mutant animals transgenic for LUC::GFP, at the YA stage. The graph shows data from four independent experimental replicates. (**d**) Total GFP fluorescence intensity from wild-type, *eat-2*(*ad1116*) and *eat-2*(*ad1113*) mutant animals transgenic for LUC::GFP, at the YA stage. The graph shows data from one representative experiment out of three biological replicates. (**e**) Bioluminescence signal of wild-type and *adsl-1*(*tm3328*) mutant animals transgenic for LUC::GFP. A time interval within the L4 stage is shown. *adsl-1*(*tm3328*) signal exhibits interruptions and reduction in the luminescence signal compared to wild-type. The graph shows data from a representative well out of 21 animals assayed in three experimental replicates. Error bars show s.d. All experiments were done using strains carrying the integrated transgene *feIs4*.

We measured luminescence from single animals that constitutively and ubiquitously express the luciferase protein fused to GFP (*Psur-5::luc*^+^*::gfp*)^23,24^, in 96-well plates. The expression of the LUC::GFP protein in this strain is driven by the regulatory region of the constitutively expressed *sur-5* gene and LUC::GFP fusion protein is expressed in most cells of *C. elegans* throughout development^23^. We transferred L4 larvae from NGM agar plates to a 96-well plate, one worm per well, containing S-basal, food (*E. coli*) and luciferin (100 μM). We did not add compounds to increase the permeability of the cuticle, so that the luminescence signal comes primarily from the ingested luciferin. The L4 larvae ingest luciferin and emit light until they reach the fourth molt (L4 to adult). During molt stages, *C. elegans* forms a plug of extracellular material in the buccal cavity and they cease pharyngeal pumping^26^ and luminescence signal drops (Fig. 1a). After the quiescent period of the molt, worms resume feeding and luciferin uptake, as the animal enters the young-adult stage, provoking an increase in the luminescence signal. We took as reference value the mean of the luminescence signal within the first 5 hours of adulthood. However, shorter measurement periods are possible.

If luciferin is limiting the reaction, differential feeding rates, and so differential luciferin uptake, will result in different luminescence signals reflecting the amount of ingested food. We tested different luciferin concentrations (from 100 to 12.5 μM) and observed that decreasing luciferin resulted in a decrease of the luminescence signal (Supplementary Fig. S1). This indicated that luciferin was limiting the luminescence reaction, at least in the tested range. We established 100 μM as the default luciferin concentration for the rest of the experiments. We also tested that luciferin can last for at least one week to ten days under our experimental conditions.

### Luminescence signal reports reduced feeding rates in mutants and detects alterations in feeding patterns

*eat-2* encodes a nicotinic acetylcholine receptor subunit and functions post-synaptically in pharyngeal muscle regulating the rate of pharyngeal pumping^27^. Consequently, *eat-2* mutants show a slow pumping phenotype, have a reduced feeding rate and are food restricted^28,29^. We crossed two pharyngeal pumping mutants, *eat-2*(*ad1116*) and *eat-2*(*ad1113*), with the LUC::GFP reporter strain. The resulting strains have reduced pharyngeal pumping rates, as expected (Fig. 1b). We therefore tested our method and measured luminescence from single animals (wild-type and *eat-2* mutants) within the first five hours of adulthood. Mean luminescence signal emitted from single *eat-2* mutants was reduced compared to wild-type animals (Fig. 1c), while no significant difference was detected between the two different *eat-2* alleles. As it was mentioned above, the reporter has the advantage of harbouring the GFP protein fused to LUC. Therefore, levels of fluorescent protein can be easily investigated, allowing the evaluation of luciferase protein levels in any strain tested and under any experimental condition. We have noticed that some metabolically compromised worms show alterations in fusion protein levels (Supplementary Table S1). To discard that the reduced luminescence observed in *eat-2* mutants could be a consequence of lower LUC::GFP fusion protein levels, we measure total GPF intensity under the same conditions of the luminescence assay. There were no significant differences in the fluorescence signals among the strains (Fig. 1d). Given that LUC catalyses the conversion of luciferin into oxyluciferin with ATP consumption^25^, it could be argued that reduction in the *eat-2* signal is a consequence of ATP depletion. However, Houthoofd *et al*. established that ATP levels for wild-type worms and *eat-2* mutants, raised on either agar plates or liquid cultures, were not affected at the young adult stage^30^. We hence concluded that the reduction in the luminescence signal emitted by *eat-2* mutants results from a specific reduction of the ingested luciferin and hence reflects reduced food intake.

We tested whether this method can be useful to monitor feeding behaviour in small populations of worms. We measured luminescence signals from 20 synchronized L1 larvae per well, in a 96-well plate, throughout larval development. Age-specific luminescence signal from *eat-2* mutant was reduced compared to the wild-type strain at all stages of development (Supplementary Fig. S2), validating the method to scrutinize feeding behaviour in populations of worms at any desired developmental stage, or through complete development. The mean luminescence signal emitted by *eat-2* mutants and wild-type animals at the L1 stage was calculated as an example (Supplementary Fig. S2).

A method that continuously reports food intake, should be suitable to identify new mutants presenting anomalous food intake patterns. Indeed, in the course of our experiments we serendipitously detected a reduced and irregular luminescence pattern with interruptions of the bioluminescence signal in the *adsl-1*(*tm3328*) mutant (Fig. 1e), thus showing a food intake defect in this mutant. *adsl-1* encodes for the enzyme adenylsuccinate lyase, involved in the purine biosynthesis pathway^31^. We confirmed this aberrant feeding behaviour by means of direct observation under the stereoscope. *adsl-1*(*tm3328*) animals showed an anomalous dynamic of pharyngeal pumping on food, with irregular rhythm of pumps, frequent long pauses and alternation between regular and slow pumping rates (in a range from 0 to approximately 140 pumps/30 sec in L4 animals; data not shown). This phenotype is difficult to characterize and quantify using short-term observation techniques and automated longitudinal technics will help the identification of irregular feeding patterns. This method represents an alternative methodology to shed light in feeding dynamics during long and continuous periods of time. We had no previous indication suggesting food intake defects in *adsl-1*(*tm3328*) mutants, confirming that our method allows the identification of new genes affecting food intake.

### Luminescence signal reports the response to changes in food availability and quality

**Figure 2:**
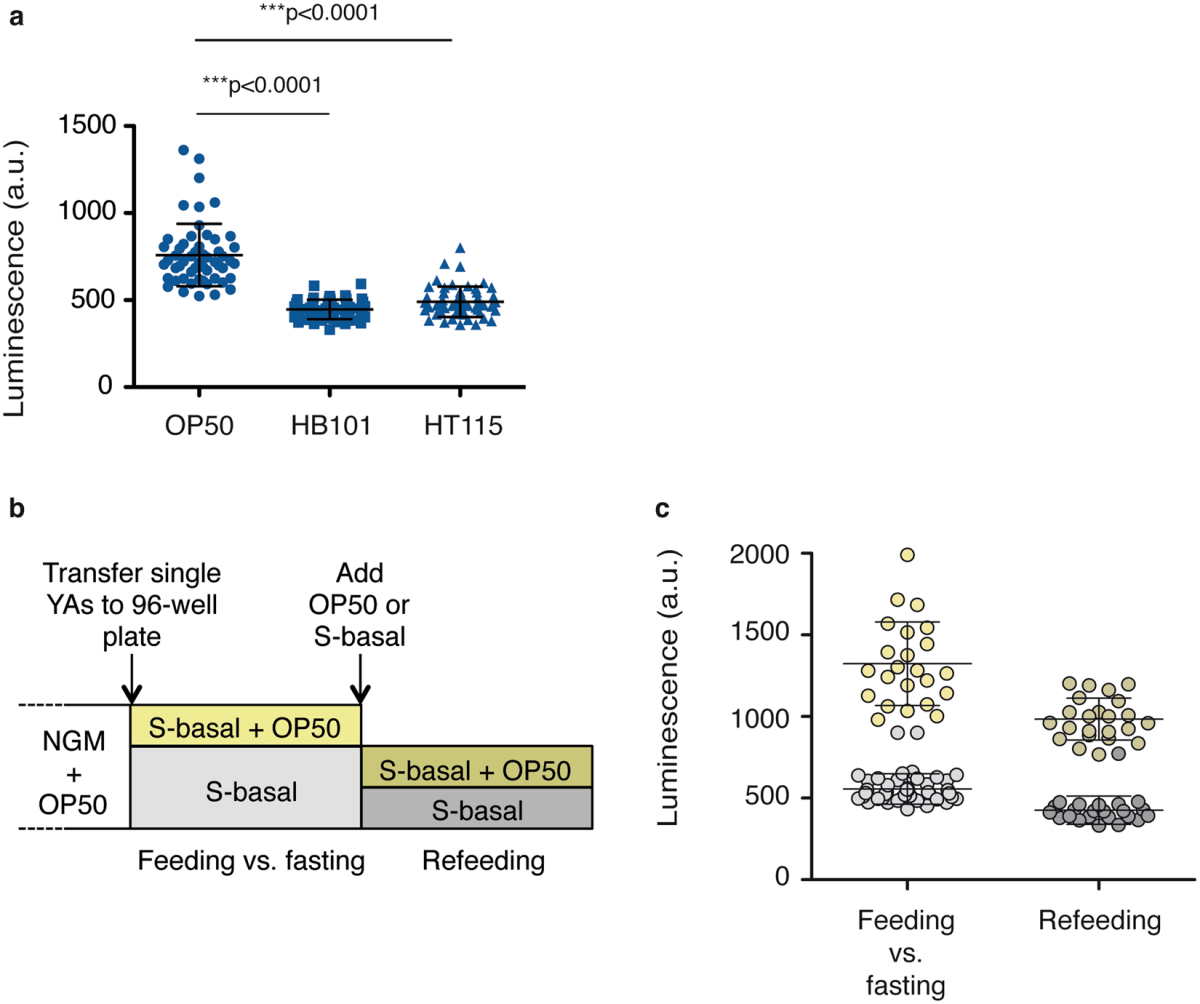
Luminescence signal reports responses to food availability and quality. (**a**) Mean bioluminescence signal from single LUC:GFP transgenic animals at young-adult stage (YA). Worms were fed on OP50, HB101, or HT115, as indicated. The graph shows data from two experimental replicates. (**b**) Description of the experiment of fasting and refeeding. YA animals are transferred from an NGM plate with OP50 to a well of a 96-well plate with 10 g/l OP50 in S-basal (Feeding). Alternatively, YA animals starved for 1 hour, are transferred to a well of a 96-well plate with S-basal (Fasting). After two hours of continuous measurement, the wells containing fasting animals are supplemented with either OP50 in S-basal (Refeeding) or S-basal alone, and are measured for another two hours. (**c**) Mean luminescence signal of two hours measurements for each condition. The graph shows data from one representative experiment of two independent replicates. The replicate is shown in Supplementary Fig. S3b. Error bars show s.d. All experiments were done using a strain carrying the integrated transgene *sevIs1*.

Environmental conditions, previous experience, food quality and the own energetic state of the animal, among others, modulate feeding behaviour in *C. elegans*^32–35^. Tipically, in laboratory conditions, *C. elegans* is fed on *E. coli* strain OP50^36^. However, it is documented that other bacterial strains are nutrient-richer food and have superior ability to support *C. elegans* growth than OP50^37–39^. Different diets induce particular behavioural responses. HB101 strain is high quality food and considered easier for worms to eat than other *E. coli* strains. Worms fed on HB101 grow faster and decrease pumping rate^38^. This feeding response is also observed in HT115, the *E. coli* strain used for RNAi assays^40^. We measured this differential modulation of food intake by distinctive food quality diets on the luminometry assay. That is, we tested if our method was able to detect the lower feeding rates reported on animals fed on HT115 or HB101 compared to OP50. Single L4 larvae, from NGM agar plates seeded with OP50, were transferred to a 96-well plate, one worm per well. Single worms were then simultaneously exposed to OP50, HB101, or HT115. Animals completed L4 larva stage on the specific diets, molted, and luminescence signal within the first five hours of adulthood was measured (Fig. 2a). The luminescence signal emitted by worms feeding on HB101 or HT115 was significantly lower than the one on OP50, as expected for lower feeding rates. Therefore, we concluded that our methodology is sensitive to detect the decreased food consumption already reported on *C. elegans* fed on HB101 and HT115.

Animals also reduce their pumping rate when deprived of food and increase it again when they encounter food^41–43^. We measured the response of *C. elegans* to changes in food availability using luminometry. We transferred single YA worms from an NGM plate with OP50 to independent wells of a plate for luminometry (Fig. 2b). When there is only S-basal in the well, the animals show a reduced luminescence signal compared to the animals transferred to wells with 10 g/l OP50 in S-basal (Fig. 2c). Upon addition of food to the fasting animals, the signal increased again, unlike in the control situation when only S-basal is added to the fasted worms (Fig. 2c). The signal over the periods of fasting and refeeding can be followed if measurements are performed at short time intervals. During continuous feeding the signal increases slowly, probably due to the increase in size of the animal. When animals are fed after a period of fasting, the signal increases to reach a similar level to that of fed worms (Supplementary Fig. S3). This assay can therefore be used to study the modulation of food intake in response to changes in food quality or other environmental cues.

### Serotonin and naloxone respectively increase and decrease the luminescence signal

To validate the suitability of the method for screening purposes or pharmacological tests, such as those on drugs that can potentially regulate feeding, we challenged our method with two compounds: serotonin and naloxone, that stimulate and inhibit feeding in wild-type worms, respectively.

**Figure 3:**
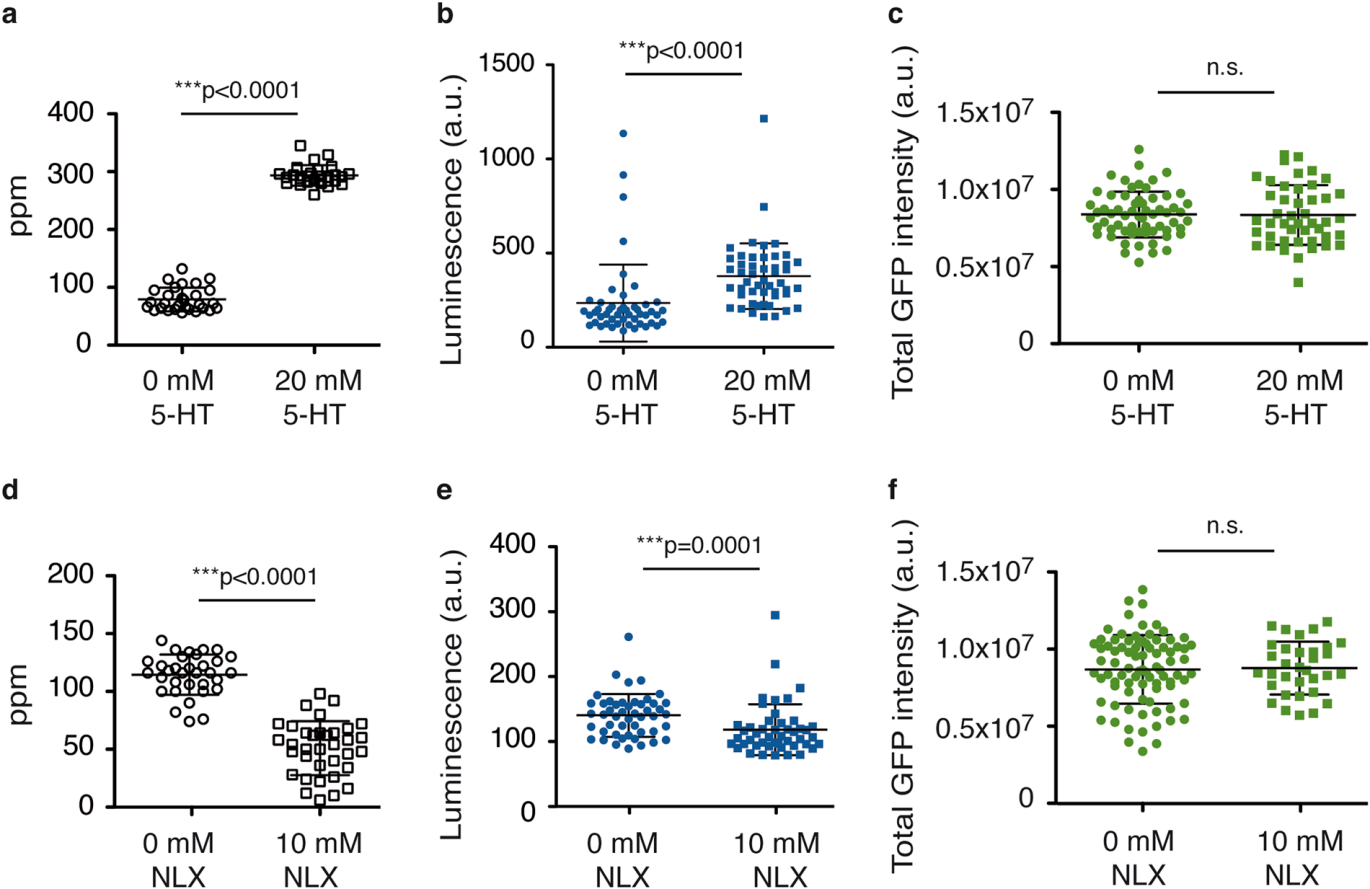
Luminescence signal respectively increases and decreases upon serotonin and naloxone treatment. (**a**) Mean pharyngeal pumping per minute (ppm) of single LUC::GFP transgenic animals, at the young-adult stage (YA), in the absence and presence of serotonin (5-HT). The graph shows data from two experimental replicates. (**b**) Mean luminescence signal from individual LUC::GFP transgenic animals, at the YA stage, in the absence and presence of serotonin (5-HT). The graph shows data from six experimental replicates. (**c**) Total fluorescence intensity from individual YA LUC::GFP transgenic animals in the absence and presence of serotonin (5-HT). The graph shows data from three experimental replicates. (**d**) Mean pharyngeal pumping per minute (ppm) of single LUC::GFP transgenic animals, in the absence and presence of naloxone (NLX), at the YA stage. The graph shows data from two independent biological replicates. (**e**) Mean luminescence signal from individual YA LUC::GFP transgenic animals in the absence or presence of naloxone (NLX). The graph shows data from six experimental replicates. (**f**) Total fluorescence intensity from individual LUC::GFP transgenic animals, in the absence or presence of naloxone (NLX), at the YA stage. The graph shows data from one representative experiment of two independent replicates. Error bars show s.d. All experiments were done using a strain carrying the integrated transgene *sevIs1*.

Serotonin is a biogenic amine neurotransmitter that modulates locomotion, egg laying and pharyngeal pumping in *C. elegans*^44^. Addition of external serotonin increases pharyngeal pumping frequency and consequently food intake^45,46^. We tested the serotonin response in our LUC::GFP reporter strain. Most studies use serotonin concentrations between 10 and 20 mM^47–49^. We set up serotonin 20 mM for all our assay conditions. The drug treatment increased pharyngeal pumping frequency of young-adult animals as predicted (Fig. 3a). We hypothesized that, if we add serotonin to the media, an increase in pharyngeal pumping would be also accompanied by an increase in the luciferin ingested and so in luminescence signal. We conducted luminescence assays in our system on serotonin treated worms. The luminescence signal of young-adult stage animals was higher in the presence of serotonin as compared to the control condition. Mean luminescence value within a 45 min period was taken as reference value (Fig. 3b). We noticed that the luminescence signal was serotonin-dose dependent and we did find that saturation of the effect was reached by the range 10–20 mM (Supplementary Fig. S4). Other authors find that this saturation was reached by 5 mM^50^. Differential experimental conditions from one study to another could explain this discrepancy. To rule out the hypothesis that serotonin was affecting protein expression levels of the LUC::GFP fusion protein, we measured total GPF intensity of serotonin-treated worms. We did not find significant differences in GFP intensity upon serotonin treatment (Fig. 3c).

Naloxone is a morphine antagonist that inhibits food intake in starved worms^51,52^. We tested the naloxone response in LUC::GFP animals and confirmed that the drug treatment reduces pumping rate under our experimental conditions (Fig. 3d). We conducted luminescence assays on naloxone treated worms. The luminescence signal of young-adult animals was lower in the presence of naloxone. As above, mean value within a 45 min period was taken as reference value (Fig. 3e). We ruled out the possibility of naloxone affecting LUC::GFP fusion protein levels by measuring total GPF intensity of the worms under naloxone treatment and did not find significant differences (Fig. 3f). We conclude that the method presented here will be useful to unravel altered feeding phenotypes under different conditions or upon different drug treatments, as it is able to detect both increases and decreases in feeding rates.

### Food intake behaviour can be detected in real time

**Figure 4:**
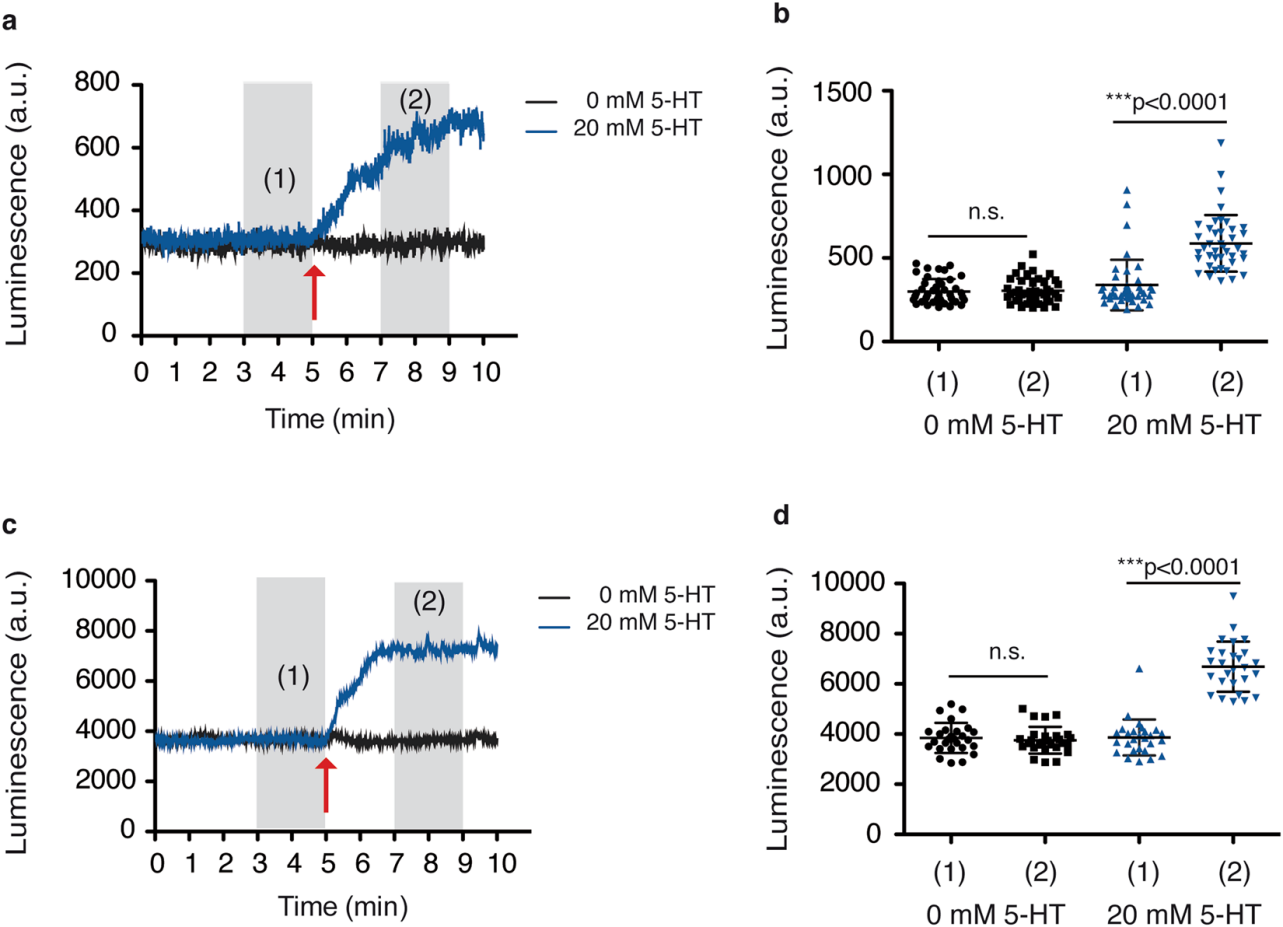
Luminescence signal reports the response to drugs on real time. (**a**) Kinetics of the luminescence signal before and after serotonin (5-HT) addition. A single LUC::GFP transgenic animal at the young-adult (YA) stage was measured. Red arrow points out the time point of 5-HT addition. (**b**) Mean luminescence signal before (1) and after (2) 5-HT addition, in single LUC::GFP transgenic animals, at the YA stage. The graph shows mean values over a 2 minutes period (grey shadows (1) and (2) shown in a) from three experimental replicates. (**c**) Kinetics of the luminescence signal before and after 5-HT addition, in a population of 20 LUC::GFP transgenic animals, at YA, per well. Red arrow indicates the time point of 5-HT addition. (**d**) Mean luminescence signal before (1) and after (2) 5-HT addition in a population of 20 LUC::GFP transgenic animals, at YA, per well. The graph shows data from two experimental replicates. Error bars show s.d. All experiments were done using a strain carrying the integrated transgene *sevIs1*.

We have shown that this method allows the study of different feeding phenotypes, particularly during medium and long periods of time. Next, we determined whether this luminescence assay is sufficiently sensitive and precise to monitor changes in food intake behaviour in real time. With this purpose, we measured the luminescence emitted by single worms at the young-adult stage, in a 96-well plate, in a kinetic fashion as follows: Luminesce signal from individual worms was measured continuously for 5 min. Then, either serotonin dissolved in S-basal (20 mM final concentration) or S-basal alone were automatically injected into the well and measurements continued for another 5 min. The signal was constant within the very first 5 min, but the addition of the drug was accompanied by an immediate increase in the luminescence signal (Fig. 4a). Only two minutes after the addition of serotonin, the signal had increased by 83.43% ( ± 39.91 s.d.) relative to the basal value before serotonin was added (Fig. 4b). Also, if required, the response rate to the treatment could be inferred from the slope of the kinetic curve. In addition, we verified that this method was also suitable to measure real time changes in food intake of small populations. We performed the same type of kinetic assay in a population of 20 worms per well. Once again, the effect of the addition of the drug was observed by a rapid increase in the luminescence signal (Fig. 4c). The increment in luminescence signal in the worm population was in the same range as in single worms (74.99% ± 20.37 s.d.) (Fig. 4d).

In summary, the assay we describe here allows the automatic and continuous analysis of food intake in a multi-well format. This method permits the scrutiny of feeding behaviours at specific time-points, during long periods of time and in real time, being suitable for both, populations and single worms at any developmental stage. This methodology is a versatile tool that might prove useful to address other feeding behaviours and patterns, such as burst feeding and pauses, food seeking and avoidance, or satiety quiescence. The method described here adds an invaluable tool to investigate how internal and external cues modulate feeding behaviours.

## Methods

### Strains and experimental conditions

We used the reporter strains PE254 (*fels4*[*Psur-5::luc*^+^*::gfp; rol-6* (*su1006*)]V)^23,24^, MRS290 (*eat-2* (*ad1116*)II; *fels4*[*Psur-5::luc*^+^*::gfp; rol-6(su1006)*]V), MRS307 (*eat-2* (*ad1113*)II; *fels4*[*Psur-5::luc*^+^*::gfp*; *rol-6*(*su1006*)]V), WBX130 (*adsl-1*(*tm3328*)/*hT2* [*bli-4*(*e937*) *let-?* (*q782*) *qls48*]; *fels4*[*Psur-5::luc*^+^*::gfp; rol-6* (*su1006*)]V), MRS382 (*sgk-1*(*ok538*)X; *feIs4*[*Psur-5::luc* + *::gfp*; *rol-6(su1006)*]V); MRS423 (*daf-16*(*mu86*); *sevIs1*[*Psur-5::luc* + *::gfp*]X); MRS433 (*daf-2*(*e1370*)III; *sevIs1*[*Psur-5::luc* + *::gfp*]X); MOL43 (*sgk-1*(*ft-15*) X; *sevls2*[*Psur-5::luc*^+^*::gfp*]); MOL62 (*age-1*(*mg305*)II; *sevIs1*[*Psur-5::luc* + *::gfp*]X). To generate MRS387 (*sevls1*[*Psur-5::luc*^+^*::gfp*]X), we X-irradiated the strain *sevEx1*[*Psur-5::luc* + *::gfp*] to integrate the reporter array. The strain was outcrossed 10 times to the N2 strain. To generate MRS389 (*sevls2*[*Psur-5::luc*^+^*::gfp*]), we X-irradiated *sevEx1*[*Psur-5::luc* + *::gfp*] to integrate the reporter array. The strain was outcrossed 10 times to the N2 strain. For stock animals we cultured the worms according to standard methods^36^. We routinely maintained the strains at 20 °C on nematode growth media (NGM) plates with a lawn of *Escherichia coli* OP50-1. Other *Escherichia coli* strains used in this work are HB101 [*supE44, hsdS20(rB-mB-), recA13, ara-14, proA2, lacY1 galK2, rpsL20, xyl-5, mtl-1*] and HT115(DE3) [*F-, mcrA, mcrB, IN(rrnD-rrnE)1, rnc14::Tn10*(DE3 lysogen: *lavUV5* promoter -T7 polymerase]. All *E. coli* strains were obtained from the Caenorhabditis Genetics Center (CGC). Synchronized worms were obtained placing 15-20 young adults laying eggs for 2–3 hours at 20 °C on NGM plates seeded with *E. coli*. Adults were removed and plates were incubated until animals reached the L4 stage or young-adult stage, as desirable. To obtain synchronized L1 larvae, we collected eggs by hypochlorite treatment and allowed them to hatch and arrest by overnight incubation in M9.

### Pharyngeal pumping assay

Mid-L4 larvae were transferred to fresh NGM plates seeded with *E. coli*, and incubated 5-6 hours until they reached the early young-adult stage. Young adults were then transferred to a new fresh NGM plate seeded with *E. coli* and allowed to crawl for 15 min. Pharyngeal pumping rate was determined by visual counting of the movement of the grinder under a dissecting microscope for 30 seconds.

### Luminometry of animals in the presence of food

Luminometry assays were performed as previously described^22^ with some modifications. Briefly, mid-L4 larvae were transferred into a well of a white, flat bottom, 96-well plate by manual picking. Plate contained 100 μl of S-basal per well. Alternatively, arrested L1 larvae were transferred into a well by pipetting. Then animals were simultaneously exposed to food by adding 100 μl of S-basal with 20 g/litre of *E. coli* OP50-1 (wet weight) and 200 μM D-Luciferin per well. Each well finally contained 200 μl S-basal with 100 μM D-Luciferin and 10 g/litre of *E. coli* OP50-1. Plates were sealed with a gas-permeable cover (Breathe Easier, Diversified Biotech). We measured luminescence (Berthold Centro LB960 XS3 equipped with two injectors) for 1 s, typically at 5-min intervals. We performed all experiments at 20 °C inside temperature-controlled incubators (Panasonic MIR-154). The raw data from the luminometer were visualized using ChronoOSX 3^53^. MS-Excel 2011 and GraphPad Prism 5 were used for all calculations and plotting of the data.

For feeding assays in presence of HB101 or HT115(DE3), well-fed mid-L4 larvae were transferred from plates seeded with OP50-1 to a fresh NGM plate without *E. coli*. Animals were allowed to crawl while incubated at 20 °C for 30 min. Then animals were transferred into a well of a white, flat bottom, 96-well plate by manual picking. Luminometer assay was performed as described above, but animals were simultaneously exposed to HB101 or HT115(DE3) as indicated. OP50-1 strain was used for control conditions.

### Luminometry of animals in fasting/refeeding assay

For fasting condition, well-fed young-adult animals were transferred to a fresh NGM plate without *E. coli* and allowed to crawl while incubated at 20 °C for 1 hour. Single animals were then transferred into a well of a white, flat bottom, 96-well plate by manual picking. Each well contained 100 μl of S-basal with 100 μM D-Luciferin. Luminescence was read for 1 sec, at 5-min intervals, for a period of 2 hours. After this period, 100 μl of S-basal with 20 g/litre of *E. coli* OP50-1 and 100 μM D-Luciferin was added to the wells for the refeeding conditions. Each well finally contained 200 μl S-basal with 100 μM D-Luciferin and 10 g/litre of *E. coli* OP50-1. For control conditions 100 μl of S-basal with 100 μM D-Luciferin was added to the wells. Luminescence was read again for another period of 2 hours. Alternatively and in parallel, for feeding conditions, single well-fed young-adult animals were transferred into a well of a white, flat bottom, 96-well plate. Plate containing 100 μl of S-basal with 100 μM D-Luciferin and 10 g/litre of *E. coli* OP50-1 per well. Luminescence was read for 1 sec, at 5-min intervals, for a period of 4 hours.

### Total fluorescence signal analysis

To measure total green fluorescence signal per worm, a young adult-stage population was washed from the plate and transferred to clear, flat bottom, 96-well plate. Worms were paralyzed with 10 mM levamisole (L9756, SIGMA-ALDRICH). Plates were sealed with a seal plate adhesive (SealMate System, Excel Scientific) and imaged in the automated microscope IN Cell Analyzer 2000 (GE Healthcare Life Science) using an objective Plan Apo 2X/0.1. Total GFP intensity was calculated with the Developer Toolbox (GE Healthcare Life Science) integrated software.

### Drug treatment assays

Serotonin (5-HT) (H7752, SIGMA-ALDRICH) and Naloxone (PHR1802, SIGMA-ALDRICH) stocks were always freshly prepared in S-basal. To assess the effect of 5-HT, synchronized young adult-stage worms were washed out with S-basal from NGM plates seeded with OP50. Animals were transferred to fresh NGM plate, in the absence of food, and incubated at 20 °C for 1 hour. Starved worms were then transferred to NGM plates, without food, containing 5-HT (20 mM) and pharyngeal pumping was score after a 15-min acclimation period. For the luminescence assay, worms starved for 1 hour were transferred into a well of a 96-well plate containing 200 μl of S-basal and luciferin (100 μM), either with serotonin (20 mM) or without for control conditions. After a period of 15 min of acclimation, luminescence was read for 0.5 sec, typically at 20-sec intervals. To measure total green fluorescence signal, 1-hour starved worms were washed and transferred to 96-well plates, containing S-basal either with serotonin (20 mM) or without for control conditions. Fluorescence signal was analysed after 15 min of acclimation period. For Naloxone treatment (10 mM) we followed the same procedure but the acclimation period was 1 hour in all cases.

For real time assays, starved worms were transferred into a well of a 96-well plate containing 120 μl of S-basal and luciferin (100 μM). We measured luminescence for 0.5 sec, continuously, for a 5-min period. Then 80 μl of 5-HT stock solution (dissolved in S-basal and luciferin 100 μM) were automatically injected (final volume in the well: 200 μl, luciferin 100 μM, 5-HT 20 mM). For control conditions 80 μl of S-basal solution with luciferin (100 μM) was added. Luminescence measurement was resumed immediately after injection and continued for another 5 min.

### Statistical analysis

Statistics were performed using GraphPad Prism 5 software. Before plotting the data, we removed the outliers detected using the Grubb’s test. Variations in luminescence, fluorescence signals and pumping rates were analysed with the nonparametric Mann-Whitney U test, two-tailed.

## Electronic supplementary material

Supplementary Information
