# Supplementary material for: An automated method for the analysis of food intake behaviour in Caenorhabditis elegans
Source: Sci Rep. 2018 Feb 26;8:3633. doi: 10.1038/s41598-018-21964-z (PMC5832146; doi:10.1038/s41598-018-21964-z)
Supplement: Supplementary file 1 — Supplementary Information [file 41598_2018_21964_MOESM1_ESM.pdf]

Supplementary Information

An automated method for the analysis of food intake behaviour in *Caenorhabditis elegans*

Mª Jesús Rodríguez-Palero, Ana López-Díaz, Roxane Marsac, José-Eduardo Gomes, María Olmedo and Marta Artal-Sanz

Figure S1

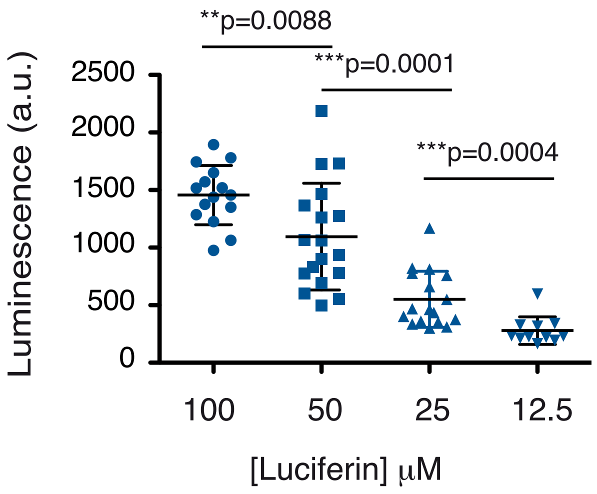

Figure S1. Mean luminescence signal from single LUC::GFP transgenic animals at the young-adult stage, at different concentrations of luciferin. Error bars show s.d. All experiments were done using strains carrying the integrated transgene *feIs4*.

Figure S2

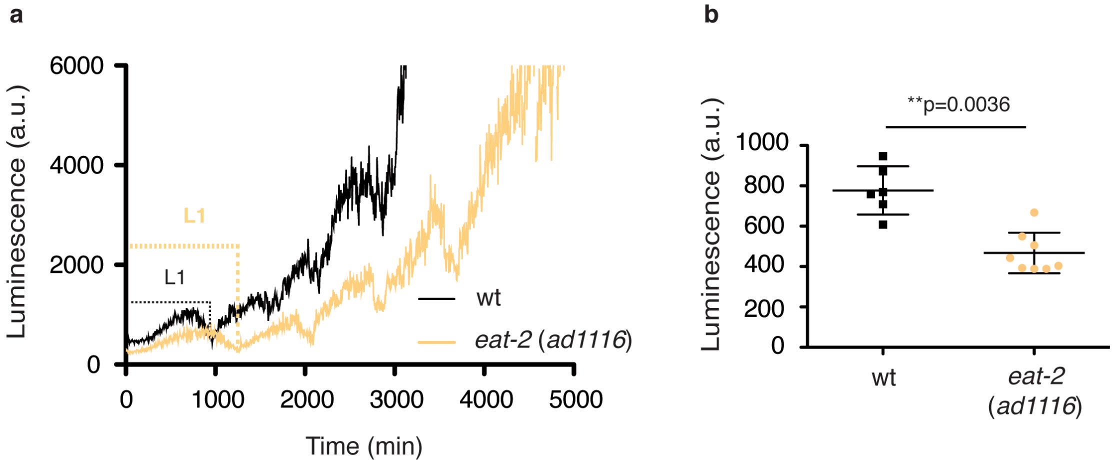

Figure S2. Luminescence signal reports food intake of populations of worms. (a) Bioluminescence signal for wild-type and *eat-2*(*ad1116*) mutant animals, transgenic for LUC::GFP, over the course of development. The graph shows data from a representative well with twenty worms. (b) Mean luminescence signal of wild type and *eat-2* (*ad1116*) population of animals, transgenic for LUC::GFP, at the L1 larval stage. Error bars show s.d. All experiments were done using strains carrying the integrated transgene *feIs4*.

Figure S3

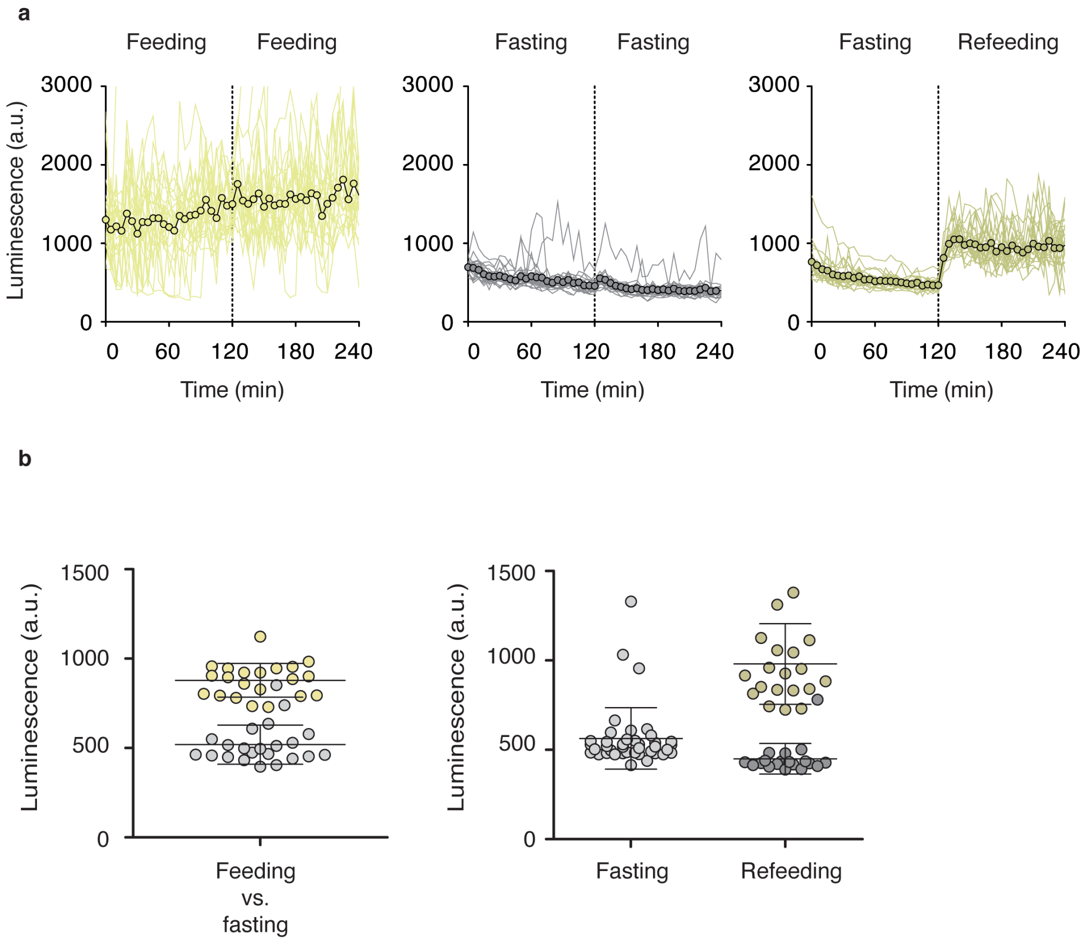

Figure S3. a) Luminescence signal over the periods of fasting and refeeding. Measurements are performed at 5-min intervals. b) Replicate of the experiment in Figure 2b. Error bars show s.d. All experiments were done using a strain carrying the integrated transgene *sevIs1*.

Figure S4

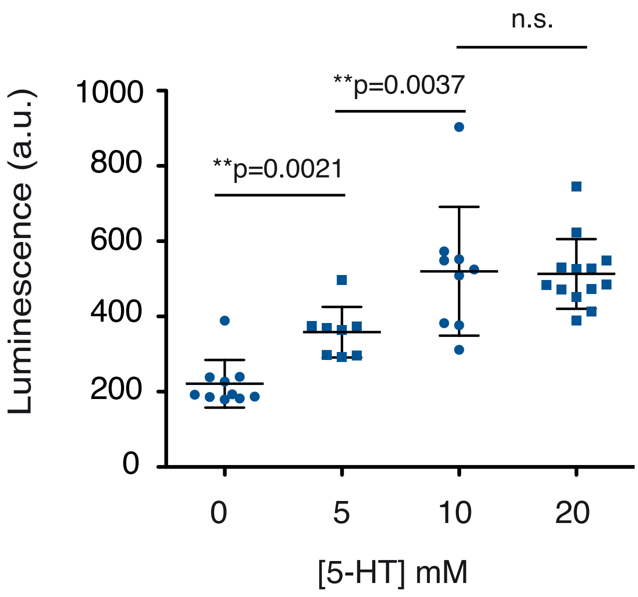

Figure S4. Mean luminescence signal from single LUC::GFP transgenic animals at the young-adult stage, at different concentrations of serotonin. Error bars show s.d. All experiments were done using strains carrying the integrated transgene *sevIs1*.

Table S1. Mean GFP fluorescence intensity in several metabolically compromised worm strains.

Strain	GFP intensity[1]	s.d.	n[2]	%[3]	P value[4]
sevIs2[*Psur-5::luc+::gfp*]	9,532E+06	1,729E+06	118	-7,93% (3)	0,0002
sgk-1(*ft-15*) X; *sevls2*[*Psur-5::luc*$^{+}$*::gfp*]	8,776E+06	1,695E+06	132		
feIs4[*Psur-5::luc+::gfp; rol-6*(*su1006*)]V	2,294E+06	6,981E+05	123	-45,47 (2)	< 0.0001
sgk-1(*ok538*)X; *feIs4*[*Psur-5::luc+::gfp*; *rol-6(su1006)*]V	1,251E+06	5,342E+05	110		
sevIs1[*Psur-5::luc+::gfp*]X	8,567E+06	1,987E+06	141	73,92 (3)	< 0.0001
daf-2(*e1370*)III; *sevIs1*[*Psur-5::luc+::gfp*]X	1,490E+07	2,730E+06	125		
sevIs1[*Psur-5::luc+::gfp*]X	7,819E+06	1,935E+06	200	11,23% (4)	< 0.0001
daf-16(*mu86*);*sevIs1*[*Psur-5::luc+::gfp*]X	8,697E+06	1,997E+06	178		
sevIs1[*Psur-5::luc+::gfp*]X	7,926E+06	2,158E+06	177	29,32% (4)	< 0.0001
age-1(*mg305*)II; *sevIs1*[*Psur-5::luc+::gfp*]X	1,025E+07	2,188E+06	91		

[1] Mean of the total GFP fluorescence intensity (a.u.).
[2] Total number of worms analyzed.
[3] Percentage of difference from the wild-type control. The number of independent replicates is shown in parentheses.
[4] P values were calculated using the nonparametric Mann-Whitney U test, two-tailed.
